# Microfluidic Mixing: A Physics-Oriented Review

**DOI:** 10.3390/mi14101827

**Published:** 2023-09-25

**Authors:** Sri Manikandan Saravanakumar, Paul-Vahe Cicek

**Affiliations:** Microtechnologies Integration & Convergence Research Group, Université du Québec à Montréal (UQAM), Montreal, QC H2X 3Y7, Canada

**Keywords:** microfluidics, micromixing, laminar flow, microdevices, vortices, lab-on-chip, electrohydrodynamics, acoustofluidics

## Abstract

This comprehensive review paper focuses on the intricate physics of microfluidics and their application in micromixing techniques. Various methods for enhancing mixing in microchannels are explored, with a keen emphasis on the underlying fluid dynamics principles. Geometrical micromixers employ complex channel designs to induce fluid–fluid interface distortions, yielding efficient mixing while retaining manufacturing simplicity. These methods synergize effectively with external techniques, showcasing promising potential. Electrohydrodynamics harnesses electrokinetic phenomena like electroosmosis, electrophoresis, and electrothermal effects. These methods offer dynamic control over mixing parameters via applied voltage, frequency, and electrode positioning, although power consumption and heating can be drawbacks. Acoustofluidics leverages acoustic waves to drive microstreaming, offering localized yet far-reaching effects. Magnetohydrodynamics, though limited in applicability to certain fluids, showcases potential by utilizing magnetic fields to propel mixing. Selecting an approach hinges on trade-offs among complexity, efficiency, and compatibility with fluid properties. Understanding the physics of fluid behavior and rationalizing these techniques aids in tailoring the most suitable micromixing solution. In a rapidly advancing field, this paper provides a consolidated understanding of these techniques, facilitating the informed choice of approach for specific microfluidic mixing needs.

## 1. Introduction

Microfluidics is a sub-domain of great interest within the realm of fluid mechanics, which has gained significant traction in various application fields, including fuel cells [1,2], microbiology [3,4,5], pharmaceutics [6,7], optics [8], and genetics. Microfluidics has revolutionized these fields by providing the ability to operate on very small sample volumes, and to manipulate fluid streams without the need for bulky mechanical transduction. While the concept of microscopic devices is already well established in microelectronics, applying similar paradigms to fluidic systems is not merely a matter of miniaturization. Indeed, modern microelectronic devices may have reached the nanoscale, but the underlying physics governing electron behavior remains unchanged (any breakthrough in quantum computing notwithstanding). In contrast, the behavior of fluids and the underlying physics radically change as the characteristic length scale of the flow contour is altered.

One of the defining characteristics of fluid flow at the microscale is its laminar behavior, thus resulting in a reduced influence of inertial forces. This paves the way for other forces, such as surface, viscous, electrochemical, and thermochemical forces, to dominate the fluid dynamics in various situations. Unlike macroscale flow, where fluid behavior and phenomena are primarily influenced by inertia and viscosity, microscale flows can be engineered to meet specific requirements and applications using alternate transduction mechanisms. This can lead to miniaturized devices without compromising the ease and efficiency of flow control.

These specific features of microscale fluid flow have been exploited for various functions, most notably in the areas of mixing, pumping, particle separation, and reactions at microscale. Exploiting external and internal factors allows for optimal utilization of microfluidic setups, as will be explored in subsequent sections of this article. Due to the dominance of surface forces and the reduced flow rates in microfluidics, managing to displace the fluid bulk from one end of the channel to the other becomes an important consideration. This can be achieved through various methods, including pressure-driven mechanisms [9,10], electrokinetic flow [11,12,13], thermal effects [14], and surface gradients. Similarly, efficient detection and separation of particles in a solution rely on the utilization of viscoelastic, inertial, and geometric effects.

The most recent developments in the field include the emergence of lab-on-chip (LoC) and micro-total analysis system (µ-TAS) devices [15,16,17] and point-of-care testing systems [18,19,20], which offer exceptional efficiency in terms of convenience, analysis speed, and sample utilization. They have proven to be vital and valuable inventions in the fields of micro-engineering, bioengineering, and genetics. Ongoing studies and research aim to create even more manufacturing-friendly LoCs, such as paper-based microdevices, lab-on-foil, and similar advancements.

Efficient micromixing is of utmost importance for LoC in particular and microfluidic devices in general, due to the laminar nature of the flows involved. For example, in a microchannel-based fuel cell, fuel-oxidant interactions are sub-optimal by default, limiting their reaction efficiency. The same limitation applies to any microfluidic system where interaction between two or more species is required. As such, effective and efficient micromixing is a topic of active research, exploring the use of various energy sources and physical transduction mechanisms. The objective of this article is to provide a review of the most recent, noteworthy micromixing techniques and approaches, with particular emphasis on the physics involved. It is the conviction of the authors that this fundamental approach will provide a better appreciation of the design parameters and trade-offs to consider when selecting a micromixing strategy. Given the unusual fluid properties at the microscale, the article begins with an essential overview of fluid dynamics in microchannels in order to provide an understanding of the governing parameters. Subsequently, the simulation models that can accurately capture specific flow characteristics are briefly addressed, offering insight into the particularities and limitations of various cases. The article then proceeds with a detailed review of state-of-the-art techniques and implementations shown to achieve efficient mixing of two miscible liquids.

## 2. Fluidics in a Microchannel

Microfluidics is an interdisciplinary field that focuses on the study of fluid flow and its properties exhibited over microstructures and microchannels. It encompasses various disciplines, including fluid mechanics, electrostatics, thermodynamics, statistical mechanics, elasticity, and polymer physics [21].

One notable difference in the fluid flow behavior of microfluidics is the diminished influence of inertial forces, which are responsible for the nonlinearity, instabilities, and turbulence commonly observed in macroscale flows. With the reduced importance of inertial effects, other parameters and factors come into play. Surface forces, in particular, exert a significant influence on the flow due to the increased surface-to-volume ratio in microfluidic systems. As the Reynolds number (Re = ρuL/μ, where ρ denotes density, u denotes velocity, L denotes the characteristic dimension, and μ denotes the viscosity) decreases, other dimensionless parameters have a substantial impact on flow dynamics.

To develop a better understanding of the field, it is beneficial to examine the effects of specific parameters on the flow mechanism through a defined contour. In this section, we explore the set of dimensionless numbers that govern and elucidate the properties of microfluidic flow. These parameters will have a strong incidence on the mechanics of micromixing. Table 1 presents these parameters and their expression.

By studying and analyzing these dimensionless parameters, we can gain valuable insight into the behavior and characteristics of fluid flow in microfluidic systems.

### 2.1. Reynolds Number (Re)

The Reynolds number is an important dimensionless number defined as the ratio of inertial forces to viscous forces, which determines the nature of the flow. In microfluidics, the value of the Reynolds number is small, due to the negligible role of inertial forces and the predominance of surface forces. A flow at the microscale is usually considered a Stokes or creeping flow, where the convective term in the Navier–Stokes equation,

(1) $$\rho \left(\frac{\partial u}{\partial t}+u.\nabla u\right)=\nabla \sigma +f=-\nabla p+\mu \left(\nabla^{2}u\right)+f$$

can be neglected, such that

(2) $$0=-\nabla p+\mu (\nabla^{2}u)+f$$

where **u** denotes the fluid velocity vector, σ denotes the Cauchy stress tensor, **f** denotes the body force vector, ρ,μ, respectively, denote the fluid density and viscosity, and *p* denotes the pressure.

This reduction of non-linearity in flow characteristics for miniaturized dimensions implies negligible turbulence and, thus, the tendency of different species to mix very slowly by default. However, in certain cases, channel geometry can reintroduce some inertial effects. For instance, in gently curved circular pipes with a radius of curvature larger than the pipe radius, a secondary flow, induced by the heterogeneous flow profile and no-slip boundary condition, can aid in micromixing. Twisted pipes have been employed to create passive serpentine micromixers [22], as subsequently discussed in Section 3.1.

While reduced inertial effects pose challenges in mixing efficiency, a small Reynolds number flow facilitates a significantly large elasticity number, which is an important parameter in rheology defined as the ratio of the Weissenberg number (*Wi*) to the Reynolds number (*Re*). It is worth noting that unlike in the macroscale, microfluidic devices allow for high deformation rates while keeping the Reynolds number as low as possible. In macro-scale devices, generating large rates of deformations and high Weissenberg numbers (which is the ratio of elastic forces to viscous forces) with low viscosity elastic fluids becomes difficult, where the induction of elastic responses is hampered, as viscoelastic effects are dampened by inertia [23,24]. The Deborah number denotes the response time of a fluid to any applied stresses normalized according to observation time, defining the rate of elastic energy being stored or released, whereas the Weissenberg number defines the effect of the elastic force over the viscous force on a fluid element.

### 2.2. Péclet Number (Pe)

In microscale mixing, molecular diffusion is the principal mechanism, with convective and advective processes potentially playing an auxiliary role. Diffusive mixing occurs at the contact interface of distinct fluid layers and is a slow process (in the order of several minutes or more), which may necessitate very long channels for sufficient homogeneity. The ratio of the rate of convection to the rate of diffusion is defined as the Péclet number, which becomes crucial in optimizing mixing in passive micromixers.

Wu et al. discuss nonlinear diffusive mixing in microchannels, where the diffusion coefficient is a function of concentration [25]. The Péclet number is a key parameter in their study, enabling a dimensionless analysis of mixing effectiveness for varying channel sizes and diffusion coefficients. To understand the significance of the Péclet number in determining mixing, let us consider the transport equation for diffusive and convective transfer, defined as

(3) $$D\left(\frac{\partial^{2}c}{\partial x^{2}}+\frac{\partial^{2}c}{\partial y^{2}}\right)=u\frac{\partial c}{\partial x}$$

Using the channel width *W*, we can obtain a dimensionless system, with x* = *x*/*W* and y* = *y*/*W* and concentration c* = *c*/$c_{0}$ – 1/2, where *D* is the diffusion constant, *u* is the velocity, and $c_{0}$ is the fully mixed solution concentration, allowing the extraction of the Péclet number, given as Pe=uW/D, from

(4) $$D\left(\frac{\partial^{2}c^{*}}{\partial x^{{*}^{2}}}+\frac{\partial^{2}c^{*}}{\partial y^{{*}^{2}}}\right)=Pe\frac{\partial c^{*}}{\partial x^{*}}$$

the analytical solution of which yields

(5) $$c^{*}\left(x^{*},y^{*}\right)=\frac{1}{\pi }\sum_{n=1}^{\infty }\exp\left(\frac{Pe-\sqrt{Pe^{2}+4{\left(2n-1\right)}^{2}\pi^{2}}}{2}x^{*}\right)\sin\left(\pi \left(2n-1\right)y^{*}\right)\left(\frac{1-\cos(\pi (2n-1)}{2n-1}\right)$$

Clearly, a larger Péclet number leads to a shorter mixing length in a channel, and denotes a stronger convective effect relative to diffusion.

The role of convection in mixing can be explained through Taylor’s dispersion phenomenon. In a circular pipe under pressure-driven (Poiseuille) flow, a thin strip element of fluid is stretched into a parabolic shape. Diffusion then occurs over the stretched strip, and after multiple iterations, the strips transform into a Gaussian distribution. Although Taylor dispersion occurs in long axial lengths and time scales, which may not in itself be practical or time-efficient for microfluidic applications, convective stretching and growth of blobs are continuously observed. Taylor dispersion cuts off the occurring convective growth and reduces it to diffusion, resulting in the evolution of pressure-driven flow as several Gaussian bands in the channel. This, in turn, increases the interfacial area for effective diffusion to occur.

In turbulent flows, the fluid elements are subject to stretching and folding, leading to an exponential increase in the contact area between species. This enhances mixing by increasing the interface area and allowing thin layers of fluids to interact with each other, whether through diffusion or convection. Similarly, the concept of flow focusing, which involves narrowing down a central flow with the aid of an outer flow, optimizes mixing time and distance.

Chaotic advection is another mixing phenomenon where the fluid elements are stretched and folded, exponentially. Even in bounded Stokes flow, chaotic mixing can occur with droplets sedimenting over a shear flow or turning microchannel. Chaotic flows can be created through various means, such as applying an electric field to induce an electrohydrodynamic advantage in droplets or a dipolar electrokinetic instability on sedimenting droplets.

### 2.3. Capillary Number (Ca)

Surface forces, particularly surface tension, play a significant role in microfluidics due to the higher surface-to-volume ratio in microchannels. Capillary forces, which describe the combined effect of surface forces and viscous forces, whose ratio is defined by the Capillary number (*Ca*), have been widely exploited in the field of microfluidics, leading to various advancements. Surface forces tend to minimize the surface energy of fluids by modifying their shape, reducing interfacial area, or causing their displacement along a surface tension gradient. On the other hand, viscous forces attempt to extend and drag the interface in the flow direction, causing instabilities and droplet formation, which has been extensively studied and applied in numerous applications.

The surface tension-driven fluid intrusion into a microchannel, with a pipe radius of w, is explained by the formation of Laplace pressure (Δp=Δγ/w), where Δγ represents the energy difference per unit area as the fluid advances into the channel, down a gradient. This leads to a pressure-driven Poiseuille flow with a velocity scale defined by u=Δγw/ηl (in a fluid column of length l, where η is the viscosity of fluid). At the microscale, fluid flow can be manipulated using two methods: solid–liquid or liquid–liquid interfacial energy. Surface tension differences can be physically created by using materials with varying surface properties (hydrophilicity or contact angle) or by generating a surface tension gradient along the flow using processes such as thermo-wetting, adding reactive fluids, self-propelling fluids/bislugs, opto-wetting, or electrowetting.

The interfacial tension depends on factors, such as temperature, surface tension gradients, and electrostatic potential. These gradients in interfacial tension induce motion at the interface, leading to Marangoni flows. The velocity profiles of flows induced by these processes are defined by u=Δγ/η, where Δγ=(∂γ/∂E)R∇E, *E* representing temperature for the thermocapillary effect and voltage for the electrocapillary effect, while *R* is the curvature scale). The most widely known Marangoni flow is thermocapillary flow.

### 2.4. Rayleigh Number (Ra)

The Rayleigh number (*Ra*) is a dimensionless number that represents the ratio between buoyancy-driven and diffusive flow in a fluid system. In the context of electrohydrodynamics (EHD), high Ra values may serve to induce EHD instability to facilitate mixing. For EHD flows, the Rayleigh number is given by $Ra_{E}=\left(\epsilon E_{0}^{2}L/\eta \right)\left(L/D\right)$, where $E_{0}$ is the applied electric field strength, ϵ and η, respectively, are the permittivity and viscosity, *L* and *D*, respectively, are the characteristic length and diameter of the channel.

In buoyancy-driven flows, diffusion may not always dominate the mixing process, and convection can then play a significant role in inducing instabilities. EHD flows exhibit similar characteristics, where the convective effects resulting from the applied electric field can lead to mixing and instabilities even when diffusion is not the dominant factor. The phenomenon of EHD instability and its implications in mixing are the subjects of ongoing research.

### 2.5. Computation Techniques

When selecting a model to simulate a microfluidic problem, it is crucial to identify the underlying physics. One important consideration for microfluidic flow computations is the point of discontinuity of the continuum approach and the region affected by the same. In microfluidics, the flow can be categorized into three major regimes based on the scale:

#### 2.5.1. Continuum

The continuum regime assumes that the continuum assumption holds throughout the flow, even at the microscale. In this case, traditional Navier–Stokes equations can be used to model the flow.

This regime is subject to a no-slip boundary condition since fluid molecules cannot penetrate through impermeable boundaries. This implies that the normal component of velocity is zero at the boundary, and the tangential component of velocity is also assumed to be zero, considering the surface-molecule interactions such as van der Waals forces, hence the Navier–Stokes equation reduces to

(6) $$0=-\nabla p+\mu (\nabla^{2}u)+f$$

with variables defined as in Equation (2). A numerical study on the micro-continuum approach using the Darcy–Brinkman approach was conducted by Carrillo et al. [26]. The model asymptotically converges to the Navier–Stokes equation and Darcy multiphase flow models. The authors tested the model by simulating capillary rise in an air-filled tube and the drainage of ethanol by air in a microchannel. The results obtained from the model are consistent with numerical solutions. In addition to the governing equations, auxiliary equations are often employed to capture complex physics in microfluidic systems. For example, many microdevices are driven by electric fields or energy, which can be described using the Poisson–Boltzmann equation or a linearized model based on Debye–Hückel theory. Elastic effects, on the other hand, are accounted for using the Oldroyd-B equations. These additional equations, along with the governing equations, are used to model and simulate various microfluidic phenomena and devices, considering the specific physics involved in each case.

#### 2.5.2. Slip Flow

In the slip flow regime, the flow behavior deviates from the continuum assumptions. In slip flow, there is a slip velocity at the fluid–solid interface, and the Navier–Stokes equations need to be modified to include slip boundary conditions. Slip flow can be simulated using methods such as the Navier–Stokes equations with slip boundary conditions or the lattice Boltzmann method (LBM).

When modeling a microfluidic setup, one of the challenges is dealing with the ambiguity of the continuum disruption at the interfaces, particularly between the walls and the flowing liquid. Various fluid–surface interaction models have been studied to address this issue. Additionally, the concept of partial slip condition introduced by Navier and Maxwell [27] can be considered and utilized for the same purpose.

The partial slip condition is expressed by the equation:

(7) $$u=\beta \left(1-\hat{n}\cdot \hat{n}\right)\left(\hat{n}\cdot \left(\nabla u+\nabla^{t}u\right)\right)$$

where $\hat{n}$ is the normal vector to the surface, **u** is the velocity vector, and β is the slip length. Another challenge arises when modeling the interface between two continuously deforming liquids (denoted by subscripts 1 and 2) with an interface velocity of **w**. The conservation of mass and a normal stress balance at the interface lead to an equilibrium equation known as Laplace’s law:

(8) $$n_{12}.\rho_{1}\left(u_{1}-w\right)=n_{12}.\rho_{2}\left(u_{2}-w\right)$$

(9) $$n_{12}(-pI+\mu_{1}\left(\nabla u_{1}+\nabla^{t}u_{1}\right)=n_{12}.\rho_{2}(-pI+\mu_{2}\left(\nabla u_{2}+\nabla^{t}u_{2}\right)+\sigma \kappa_{c}$$

where σ denotes the surface tension, ρ,μ denote the fluid properties (density and viscosity), *p* and **u**, respectively, denote pressure and velocity, and κ denotes the curvature of the interface. Regarding species or solute transport, an advection–diffusion equation can be employed:

(10) $$\frac{\partial C_{i}}{\partial t}+\nabla \cdot \left(uC_{i}\right)=\nabla \cdot \left(D_{i}\Delta C_{i}\right)$$

with $C_{i},D_{i}$, and **u** denoting the concentration, diffusivity, and velocity of the species. Thermal diffusion and electrostatic gradients can also be considered as complex forms of solute transport.

#### 2.5.3. Molecular

In this regime, the flow behavior is dominated by molecular interactions. At this scale, the continuum assumption completely breaks down, and alternate methods are required to model the flow accurately.

The fluid must be modeled as a collection of individual molecules rather than continuous bulk. In this case, specialized algorithms and methods are used to simulate the behavior of the fluid. Some popular approaches include the lattice Boltzmann method, direct simulation Monte Carlo (DSMC), stochastic rotational dynamics (SRD), and dissipative particle dynamics (DPD).

The lattice Boltzmann method is a mesoscopic approach that combines the discretization of space and time with a simplified kinetic model, and is widely used for simulating fluid flow at the microscale. The study by Hoseinpour et al. demonstrates the application of the lattice Boltzmann method to investigate the influence of viscosity and capillary number on droplet formation in a LoC T-Junction [28].

Direct simulation Monte Carlo (DSMC) is a particle-based method that simulates the behavior of individual molecules and their collisions. It is particularly suitable for modeling rarefied gas flows and can handle flow regimes where the continuum assumption breaks down. Kannan et al. studied the effects of a stationary particle on a microchannel Poiseuille flow using the DSMC method, providing insights into the Knudsen paradox, whereby molecular models can be purely stochastic or purely deterministic [29].

In microscale simulations, mesoscale models can be used to bridge the gap between the molecular and continuum approaches. Indeed, they combine a molecular description at the boundaries, where the fluid interacts with solid surfaces, and a continuum description in the bulk. However, achieving a perfect overlap between the two approaches can be challenging due to the mismatch in time scales, making accurate simulations difficult to realize.

Ultimately, the selection of the appropriate simulation model must take into account the Knudsen number and the corresponding flow regime. The review by Bazaz et al. provides a detailed overview of computational methods for inertial microfluidics, including asymptotic solutions, Navier–Stokes approaches, and the lattice Boltzmann method (LBM) [30].

### 2.6. Mixing at the Microscale

Effective micromixing is a crucial consideration for a large number of microfluidic systems, especially when they involve chemical reactions between different species, precipitation of particles, or any general interaction between distinct fluids, which is generally the case for LoC and µTAS systems. Hence, the challenge lies in the efficient combination of two fluids in both limited space and time, while operating in a laminar flow regime. Fluidic mixing can occur through diffusion, which is typically slow, or advection, which requires the generation of disturbances or agitation in the fluid.

Various mixing techniques have been explored to enhance micromixing efficiency in microfluidic devices. These techniques make use of different forces and phenomena to control flow and promote effective interaction between fluids. Some of the prominent micromixing techniques include pressure gradients [31,32,33], capillary effects (thermocapillary and electrocapillary) [33,34], electrokinetics [35,36], magnetohydrodynamics (magnetic field, Lorentz forces) [37,38], rotations, centrifugal forces, and acoustic streaming [39,40].

According to Fick’s Law, the flux of a fluid through the interface of a liquid–liquid mixture is proportional to the concentration gradient of the fluids, with the proportional constant defined as the molecular diffusivity (*D*) [41]. The transport equation for the concentration of the mixture (*c*) incorporates this diffusion process:

(11) $$u\frac{\partial c}{\partial x}=D\left(\frac{\partial^{2}c}{\partial y^{2}}\right)$$

with variables defined the same as in Equation (3).

**Figure 1:**
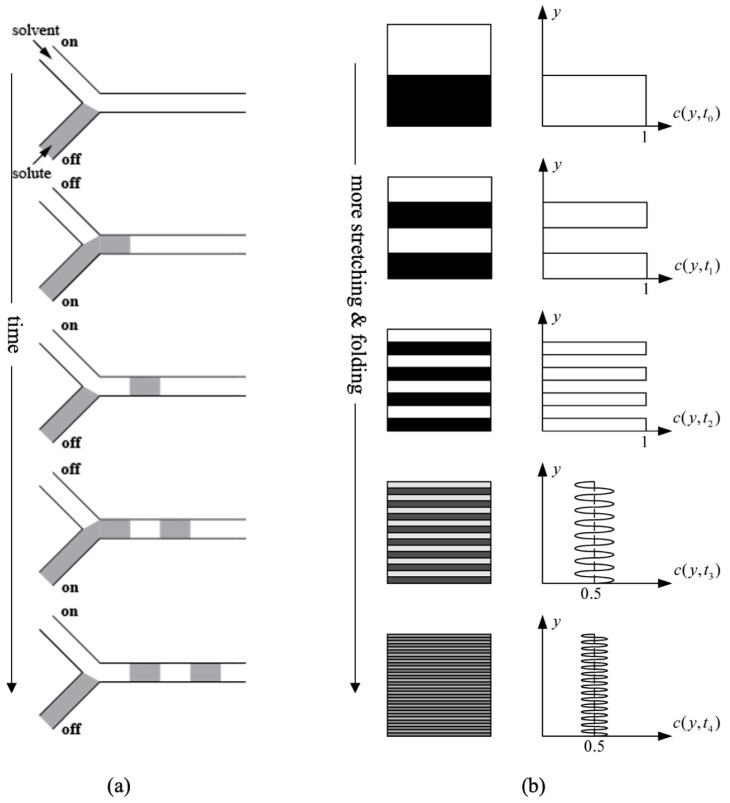
Mixing mechanisms: (**a**) Time-interleaved process. Inspired from [42] (**b**) Chaotic advection combined with molecular diffusion process showing striation pattern (left) and concentration distribution (right). Both mechanisms highlight how chaotic advection can effectively increase the contact area between the mixing species and, thus, enable diffusion to occur significantly faster. Reproduced with permission from [44].

Full mixing (characterized by a homogeneous concentration of the mixture) by means of diffusion alone, often requires a significant amount of time. To expedite the mixing process, two main methods are commonly employed, as illustrated in Figure 1. The first is time-interleaved segmentation, which involves alternately channeling the mixing liquids to increase the interfacial area for faster diffusion. This approach reduces mixing time, for *n* merging fluids, by a factor of *n*^2^ compared to the case with the original streams merely merging directly into a common channel [42].

The second method is chaotic mixing or advection [43]. Chaotic mixing involves stretching and folding fluid blobs, leading to the exponential reduction of striation thickness. In fact, this approach enhances mixing efficiency by promoting faster piecewise diffusion. Suh et al. provide detailed calculations for mixing times in various scenarios, comparing generic mixing, hydrodynamic focusing, and chaotic advection [44]. The results demonstrate significantly accelerated mixing with chaotic advection compared to other techniques.

In addition to time-interleaved segmentation and chaotic advection, forming vortices in the bulk of the fluid is another effective method for enhanced micromixing in a microchannel. Vortices can be induced by inducing local differences in fluid properties, such as viscosity, density, or thermo-chemical properties. Mechanical diffusers or barriers can also be used to induce vortices by disrupting the flow.

In practice, micromixing efficiency can be evaluated using flow visualization techniques. The mixing index (*MI*) can serve to quantify the efficiency of a micro-mixer by calculating the ratio between the concentration of species (illumination intensity of each pixel during experimentation) over the cross-section of the channel and the mean value [45]:

(12) $$MI=1-\sqrt{\frac{1}{N-1}\sum_{i=1}^{N}{\left(\frac{c_{i}-c_{av}}{c_{av}}\right)}^{2}}$$

where $c_{i}$ is the concentration at a particular point and $c_{av}$ is the average concentration of all selected points in a concentration contour perpendicular to the flow. An *MI* of 1 corresponds to perfect homogeneity.

## 3. Micromixing Techniques and Micromixers

Micromixers play a vital role in LoC and biomedical applications. Their performance parameters primarily revolve around the speed and efficiency of mixing, especially when dealing with high-viscosity fluids and low Reynolds numbers. Diffusive mixing is the key mechanism in micromixers, and optimizing it involves factors, such as the interfacial area, molecular diffusivity, and concentration gradients.

Micromixers can be categorized into passive and active types based on the usage of external energy to facilitate mixing. Passive micromixers achieve mixing without any external energy input, while active micromixers utilize various forms of energy, such as electric fields, ultrasonic waves, or magnetic forces. The design of micromixers aims to optimize the mixing length to achieve quick and efficient mixing within a small space. Additionally, the selection of materials for microchannel walls, based on their hydrophilicity, can be used to induce secondary flows and recirculation, further enhancing mixing efficiency.

**Figure 2:**
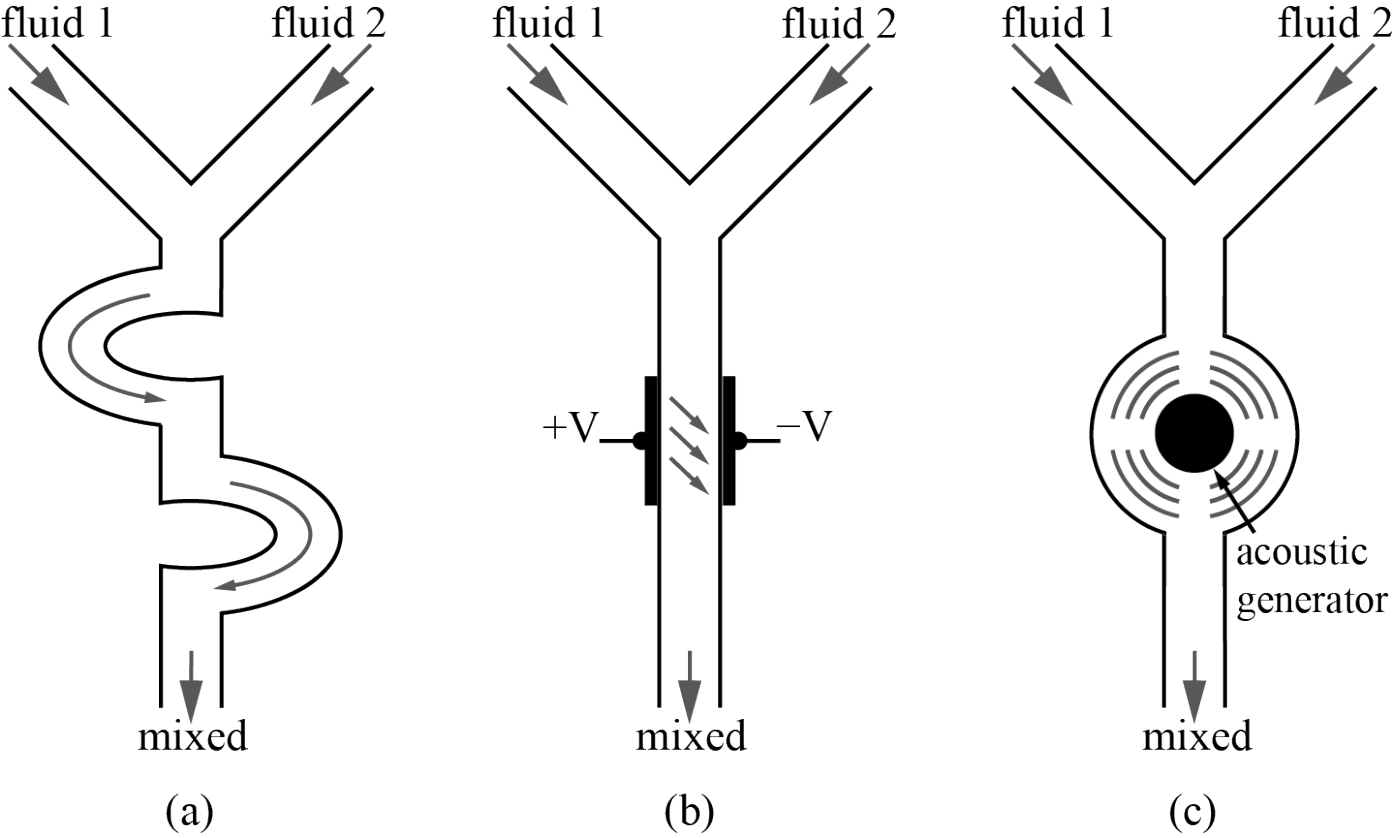
Main micromixing approaches: (**a**) Geometry-assisted: passive mixing through the channel layout and obstructions; (**b**) electrokinetic: active mixing through flow reduction by means of electric fields; (**c**) acoustic: active mixing through turbulence generation by means of mechanical actuation through the production of sound waves.

As illustrated in Figure 2, this section details the major approaches for passive micromixing, followed by two highly exploited and efficient active micromixing techniques: electrokinetic and acoustic micromixing. This article also provides an overview of other techniques and combinations of different methods that have emerged as novel designs in recent literature.

### 3.1. Geometry-Assisted

Geometry-assisted micromixers are a significant subset of passive micromixers that rely solely on channel geometry and fluid properties without the need for external energy sources. Understanding these micromixers is crucial, as they form the foundation of the field and provide insight into the underlying physics of mixing. By leveraging the advantages of channel geometry, these micromixers employ concepts, such as hydrodynamic focusing, Taylor’s dispersion, chaotic advection, stretching and folding of fluid blobs, and inducing secondary flows to enhance mixing.

One example of a geometry-assisted micromixer is the laminated micromixer, which utilizes hydrodynamic focusing. In this design, multiple reservoirs containing two different fluids are arranged in a parallel lamination configuration. The fluids are directed into a main channel through smaller channels, maintaining their laminar flow profiles. This configuration increases the interfacial contact area between the two fluids, promoting efficient mixing through diffusion.

**Figure 3:**
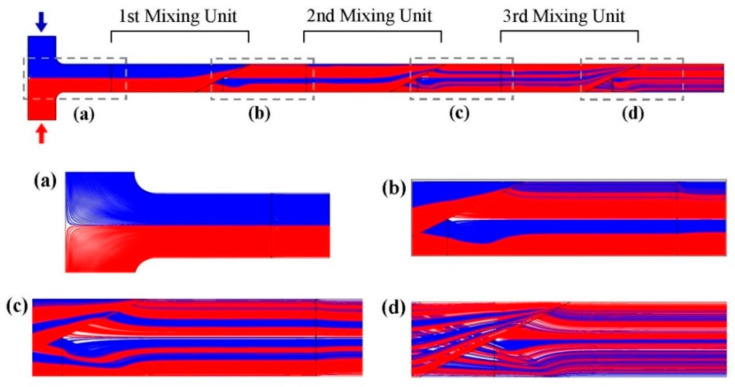
Generation of substreams through serial lamination, highlighting dynamics in (**a**) T-inlet, (**b**–**d**) first/second/third mixing units. The mixing improvement for additional mixing units is readily apparent Reproduced with permission from [46].

Another technique is serial lamination, where the streams split and join both horizontally and vertically in a three-dimensional (3D) space. This configuration enhances mixing by utilizing gravity to create disturbances or rupture interfaces as the fluid falls down the vertical sub-channels. Serial lamination has an advantage over parallel lamination as it benefits from the assistance of gravity. However, it requires higher power consumption from the pump to raise the streams above the vertically ascending sub-channels before they fall and merge with the mainstream. This concept is known as the split and recombine model [46,47,48], where a single stream splits into sub-streams in 3D or 2D space and recombines iteratively until sufficient mixing is achieved, as presented in Figure 3.

Hydrodynamic focusing mixers, while effective for enhancing mixing, can become complex in design due to the staggered placement of channels and the high number of directing channels for each fluid. Analytical solutions for mass transport in hydrodynamic focusing mixers have been investigated [49]. The mixing length in these devices is influenced by the viscosity ratio and Péclet number. It has been observed that decreasing the height-to-width ratio of the channel cross-section and increasing the sheath-to-sample flow rate ratio result in an increase in the overall mean velocity. Furthermore, increasing the Péclet number effectively reduces the mixing length, although the impact becomes insignificant beyond a certain value (typically 10).

Another mixing technique involving hydrodynamic focusing and time-interleaved segmentation aims to reduce the axial mixing path by utilizing time-interleaved segmentation [42]. The process involves switching the input of solute and solvent at a given frequency to form segments within the focused stream. Two middle inlets alternate in operation, creating segments within the focused stream. Sheath streams are inputted via the other two inlets, effectively hydrodynamically focusing the streams and reducing the transverse mixing length. Increasing the switching frequency leads to shorter segments and faster mixing, taking advantage of Taylor’s dispersion. The proposed technique aligns with the Taylor–Aris dispersion theory, as discussed in Section 2.2. Along the axial direction, the focused stream, flowing down the pressure gradient together with the sheath flow, stretches the segments due to the Poiseuille nature of the flow. This interaction increases the interfacial area, resembling Taylor’s dispersion. The dominant effect of Taylor’s dispersion in the axial direction leads to a significantly larger mixing effect compared to molecular diffusion alone. This observation is supported by the finding that the effective diffusion coefficient in the axial direction is 1815 times greater than that in the transverse direction, where only molecular diffusion is present.

The design of micromixers incorporates various elements to promote efficient mixing, including the use of T-channels, Y-channels, bends, blocks, grooves, hindrances, and multiple crossover junctions. These features create disturbances in the laminar flow, inducing chaotic advection and secondary flow within the channel [50,51]. For example, expansion–contraction cavity arrays, such as the staggered herringbone mixer (SHM), slanted groove mixer (SGM), and barrier-embedded mixer (BEM), introduce spatial perturbations and create secondary flow patterns to enhance mixing efficiency [52,53,54,55]. The use of slanted grooves in the SGM generates a short-pitched spiral flow, improving mixing efficiency [55]. Further studies on expansion–contraction (EC) cavity arrays have been conducted [56,57,58]. The study by Firmino et al. focuses on the design of a 3D micromixer for nanoliposome synthesis [59]. The microchannel in this micromixer is intentionally twisted to induce chaotic advection, which is beneficial for efficient mixing. In that design, a central stream containing lipids solubilized in dry ethanol is surrounded by two lateral streams of ultra-pure water. As the fluids flow through the twisted microchannel, the hydrodynamic compression caused by the lateral streams results in the generation of chaotic flow patterns, which disrupt laminar flow and promote thorough mixing of the lipids and water.

**Figure 4:**
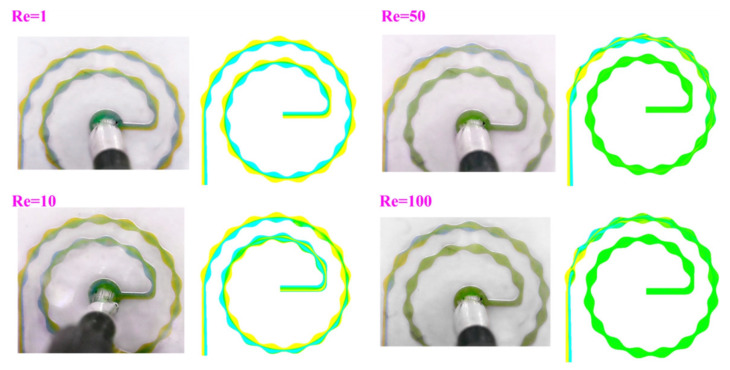
Micromixing using spiral microchannels with sinusoidal walls, shown for varying Reynolds numbers. Reproduced with permission from [64].

Dean flow (or Dean vortices) is another effective approach used to enhance mixing efficiency. Dean vortices occur in channels with bends, creating swirling flow patterns that contribute to better mixing [22,60]. Bhunia et al. classify Dean vortices into corner vortices and base vortices. At high Dean numbers, base vortices split into SingE (swallowing engulfment) and SSingE (swallowed engulfment) [61]. The presence of Dean vortices significantly affects the mixing characteristics of the flow. Peng et al. study the behavior of dean vortices for two miscible fluids in a T-micromixer [62], while Tripathi et al. study the strength of Dean flow and mixing characteristics in 3 spiral micromixers with variations in structure dimensions [63]. It is observed that, for inelastic viscous fluids, the strength of Dean flow is appreciably enhanced. Bahrami et al. demonstrate that using spiral microchannels with sinusoidal walls decreases channel length and increases mixing efficiency by creating separation vortices, as shown in Figure 4 [64]. The transition from toroidal to helical channels, as studied by Herros et al., led to a breakage of chiral symmetry in the pair of Dean vortices and a decrease in entropy production [65].

Serpentine micromixers, characterized by their simplicity and efficiency in invoking the Dean phenomenon, are widely used. Wang et al. studied serpentine micromixers with elliptical curves, further exploring the potential of this design for efficient mixing [22].

### 3.2. Electrokinetics

Electroosmosis (EO) is a key phenomenon in electrokinetics that plays a significant role in microchannel mixing. It involves the application of an external electric field perpendicular to a charged surface in an electrolytic solution. This electric field applies a net force on the fluid, inducing fluid movement or disturbance in the bulk. EO can be implemented using various charge or current input types, including alternating current electroosmosis (ACEO), induced charge electroosmosis (ICEO), and direct current electroosmosis (DCEO).

The ACEO is commonly used at low frequencies and with fluids of low conductivity, typically with electrolyte concentrations less than 0.01 mol/dm^−3^. However, the ACEO may experience limitations such as electrode degradation due to bubble formation. To remedy this, alternative approaches are explored such as ICEO and nonlinear electroosmosis.

AC electrothermal (ACET) actuation is another intriguing concept in electrokinetics. It involves forming a temperature gradient, either through external or internal means, within the fluid. This temperature gradient leads to variations in the fluid’s conductivity and dielectric constant, which, in the presence of an external AC electric field, induces stirring and mixing (in short, instabilities) in the microchannel. ACET is particularly useful for fluids with higher conductivity, as it generates strong microflows that facilitate effective mixing. The frequency range for ACET is wide, unlike ACEO. However, it may be less effective in very narrow channels due to limited volume, as it relies on bulk fluid properties to create the necessary electrical property gradients.

Electroosmosis dates back two centuries to the work of Ferdinand Friedrich Reuss who observed water movement through a clay plug when an external electric field was applied, which confirmed the discovery of both electroosmosis and electrophoresis [66]. The electric double layer (EDL) formed near the solid–liquid interface is responsible for electrokinetic phenomena. This EDL forms due to the attraction of counter ions toward a charged interface. Various models have been developed to explain the theoretical aspects and the physics underlying electrokinetic flows.

One of the early models was proposed by Hermann von Helmholtz, who suggested that the charge at the interface is balanced by ions redistributed in a plane parallel to the interface, forming the EDL [67]. That model, however, did not take into account the thermochemical properties of ions or the kinetic theory of ions. Gouy–Chapman later introduced a model that accounted for the thermal motion of ions, where the concept of a diffused layer was introduced, where ions are spread out in the vicinity of the charged interface [68,69]. The Gouy–Chapman EDL (GC EDL) model is based on the Poisson equation:

(13) $$\nabla^{2}\psi =-\frac{\rho_{e}}{\varepsilon_{0}\varepsilon_{r}}$$

where $\psi ,\rho_{e},\varepsilon_{0},\varepsilon_{r}$, respectively, denote the electric potential, ionic density, electrical permittivity of the vacuum, and relative electrical permittivity. The ionic species in the solution are affected by the electrostatic potential of the charged interface, leading to the introduction of the Boltzmann equation

(14) $$c_{i}=c_{i}^{b}\exp\left(-z_{i}\psi /k_{B}T\right)$$

where $c_{i},c_{i}^{b},z_{i}$, *T*, respectively, denote the concentration of the *i*^th^ ionic species, concentration of ions in bulk, ionic valence, and absolute temperature, and $k_{B}$ denotes the Boltzmann constant. The prior two equations together form the Poisson–Boltzmann equation, which, through the Debye–Hückel approximation, can be rewritten as

(15) $$\nabla^{2}\psi =\underset{\kappa }{\underset{︸}{\frac{2e^{2}c_{i}^{b}}{\varepsilon_{0}\varepsilon_{r}ek_{B}T}}}\psi$$

where κ is referred to as the Debye length or the characteristic length of the EDL. Many complex models have been devised following the GC EDL model. The GC EDL model also presents the drawback of not being applicable for every case of charged solid–liquid interface. The boundary between the immobile charged layer and diffused ionic layer is assumed to be the shear plane and the potential of this plane is denoted as Zeta potential. If the zeta potential is increased to a very high value, the ionic concentration value shoots up to infinity, as the model considers the ionic species to be point charges ignoring their Stokes radii and the hydrated radius in the fluid medium. Thus came the new model from Otto Stern, the Stern model, i.e., the combination of both Helmholtz and Gouy–Chapman models. The Stern model proposed that the first layer was an immobile layer of hydrated ions (as in the Helmholtz model) and the next layer was the diffused layer (as in Gouy–Chapman model) [70].

After having considered the fundamental concepts of EDL, let us explore how it can be used to manipulate fluids to cause mixing in a microchannel. The application of an AC electric field to a microchannel induces an electro-osmotic flow (EOF). To determine the velocity and other parameters related to EOF, the Navier–Stokes equation in one dimension for Stokes flow can be considered:

(16) $$\frac{\partial u}{\partial t}=\nu \left(\frac{\partial^{2}u}{\partial y^{2}}\right)$$

where *u*, *t*, *y*, ν, respectively, denote EOF velocity, time, distance normal to the channel walls, and kinematic viscosity of the fluid. At the walls, one should consider a slip boundary condition with $U_{slip(y=0)}=U_{slip(y=d)}=U_{HS}e^{iwt}$, where $U_{HS}$ is the Helmholtz–Smoluchowski velocity that is solved for the Navier–Stokes equation in the presence of the external applied electric field $E_{x}$, for a thin EDL case and no-slip channel walls [71].

The general Navier–Stokes equation for an incompressible fluid when an external electric field is applied is

(17) $$\rho \left(\frac{\partial u}{\partial t}+u.\nabla u\right)=-\nabla p+\nabla \left(\nu \left(\nabla \left(\rho u\right)\right)\right)-\rho_{e}\left(\nabla \phi +\nabla \psi \right)+\nabla P$$

where ∇ϕ is the external applied electric field, ∇ψ is the internal electric field caused by the distribution of ionic species, and *P* is the body force density due to the applied pressure gradient. The mixing phenomenon can be solved and interpreted by iteratively solving the Navier–Stokes equation along with the advection–diffusion and Poisson’s equations. Interestingly, EOF can also be extended to non-Newtonian fluids [72,73] and viscoelastic fluids [74], the effects of which alter the Navier–Stokes equation, based on inputs from modified Cauchy equations and the stress tensor.

Electrokinetic micromixers have been extensively studied to induce mixing in microchannels. Various designs and configurations to achieve efficient mixing using electroosmosis (EO) and AC electrothermal (ACET) phenomena have been attempted.

**Figure 5:**
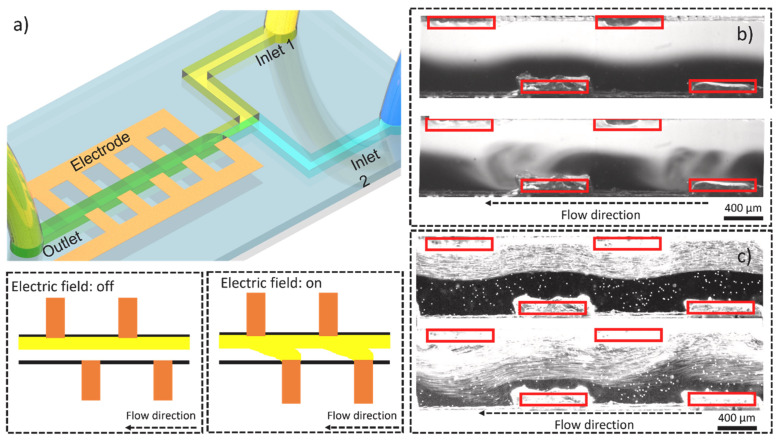
Schematic illustration of AC electroosmosis micromixing. (**a**) Device overview and working mechanism; (**b**) mixing of DI water with fluorescent dye. One notes the primary and secondary mixing patterns arising in response to applied potential. (**c**) Mixing of DI water with fluorescent particles. Reproduced with permission from [45].

Wu et al. developed a simple yet effective micromixer with a staggered arrangement of electrodes throughout the channel as presented in Figure 5 [45]. Mixing efficiency was explored in relation to varying AC frequency, flow rate, tooth number, and voltage amplitude. The observed bulk movement of fluid from one electrode to another in the transversal direction of the channel led to the mixing of two fluids. A lower frequency range (e.g., 10 Hz) was found to work best, as higher frequencies altered the field direction before the EDL had time to fully develop, weakening the effectiveness. Different EOF patterns were observed at lower flow rates, contributing to enhanced mixing compared to higher flow rates.

Feng et al. designed a 3D electro-osmotic micromixer with a rectangular conductor resembling a Rubik’s cube in the middle of the channel [75]. Each surface of the cube was divided into 9 cubes that could rotate freely, similar to a physical Rubik’s cube. By rotating different portions of the cube to control the flow, the combined effect of tube torsion and time-varying cosine voltage led to a maximum efficiency of 98.89%.

Xiong et al. investigated electro-osmotic mixing using a cantor fractal structure with three units, where each unit had a pair of electrodes at the start [76]. They combined the effects of secondary flows with electro-osmotic flow and studied the mixing by varying the direction of the electric field for each pair of electrodes. Similarly, Wu et al. explored a micromixer with a Cantor fractal structure but focused on changing the positioning of the electrodes instead of altering the field direction [77].

In the design of electro-osmotic micromixers, key parameters include the orientation of the electrodes, the frequency and amplitude of the applied voltage (for AC), and the spacing between electrode pairs, as these factors influence the formation of vortices and the interference effects between them.

**Figure 6:**
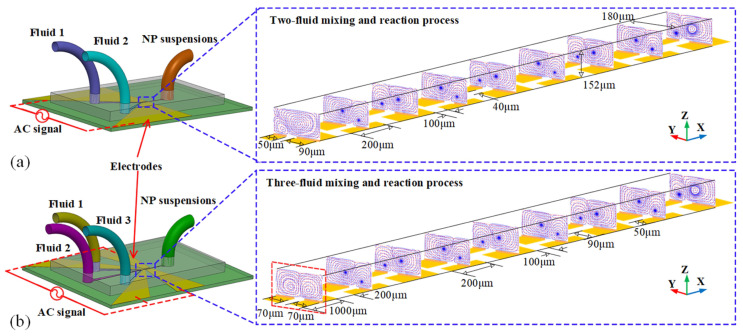
Schematic illustration of ACET-based micromixing-assisted nanoparticle (NP) synthesis. (**a**) Mechanism of two-fluid mixing and reaction based on staggered asymmetric ACET-based microvortex pairs; (**b**) mechanism of three-fluid sequential mixing and reaction process. Reproduced with permission from [81].

ACET relies on the formation of a temperature gradient within the bulk of a fluid, which in turn causes local discrepancies in the electrical properties of the fluids, especially conductivity and permittivity. These induce localized fluid circulation causing a spatial perturbation leading to the mixing effect. Furthermore, the change in electrical properties generates a charge density. On application of an external field, a force is applied over the charge density. When the charges are set to motion they also set the medium into motion, thus creating microflows and vortices leading to the mixing of fluid streams. From the works of Ramos et al. [78], and Salari et al. [79], it can be stated that the body force, hence also the velocity of microflow, is proportional to the fourth power of the applied field. However, in certain applications, strong electric fields may harm biofluids and trigger undesirable chemical reactions. Similarly, at higher temperatures, buoyancy forces electrokinetic force, thus weakening the potential effect of ACET [80]. The ratio of electrothermal force to buoyancy force is influenced by the gradient of temperature and the change in temperature (∇T/ΔT). As an alternative, external heating devices can be used to introduce temperature gradients and facilitate control over fluid flow direction. ACET concepts have been successfully employed in building various micromixer models and have proven effective for mixing fluids where EOF is unsuitable, as highlighted in Figure 6 [81,82,83].

In addition to its use for micromixing, the field of electrokinetics, including electroosmosis, AC electrothermal, electrophoresis, dielectrophoresis, and electrowetting, finds wide applicability in particle separation, particle synthesis, flow control, and other micro- and nano-scale applications.

### 3.3. Acoustic Micromixing

**Figure 7:**
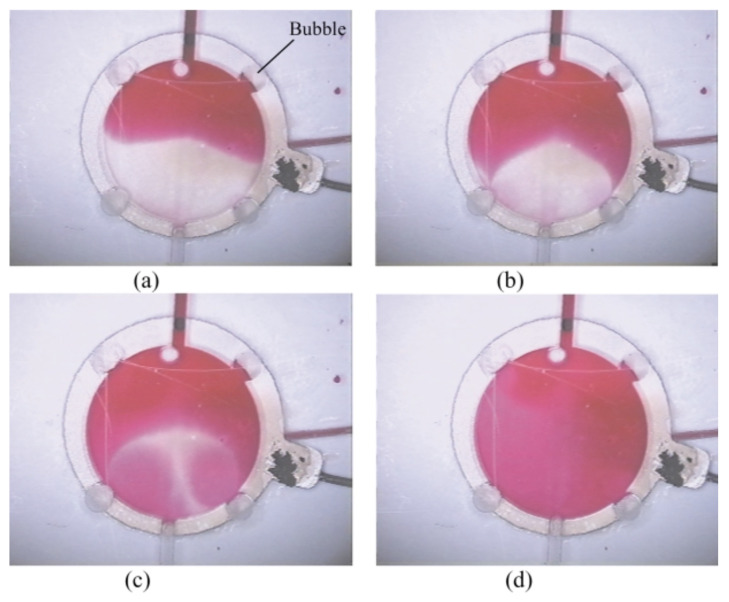
Photographs showing two fluids mixing through acoustic microstreaming in a chamber that has four air pockets (bubbles) at time (**a**) 0 s; (**b**) 10 s; (**c**) 25 s; (**d**) 45 s. An actuating piezoelectric PZT disk is attached on the backside of the chamber (not seen here). Reproduced with permission from [85].

Acoustic-driven micromixing has gained significant attention and offers interesting possibilities for achieving efficient mixing in microchannels. There are two classes of oscillating objects used to enable this approach: microbubbles [84,85,86] and microsolids [87,88]. Microbubbles, as shown in Figure 7, can generate perturbations in the fluid space but have certain disadvantages such as the existence of bubbles themselves, non-uniform bubble size, heat deformation, and unstable bubble trapping. Researchers have worked on overcoming these challenges. For instance, Ahmed et al. use horseshoe structures in the fluid passage to stabilize bubbles, while Dean vortices in a porous membrane can also be employed to generate microbubbles [89].

Microsolids have found more widespread use in acoustic-driven micromixing, although the vortices they produce are often more organized and less chaotic. To enhance mixing, structures can be installed in such a way to create interference between vortices. Sharp-edged structures, such as cantilever beams, have been considered by Huang et al. [90], where the sharp edges generate centrifugal forces that improve mixing. It is preferable to have structures with low rigidity so as to produce maximum possible disturbance for a generated amplitude of wave. Ghorbani Kharaji et al. conducted a numerical study on a sharp-edged acoustic micromixer in a microchannel, exploring parameters, such as the tip angle and sharp edge height to enhance mixing [91]. An array of structures with sharp tips positioned in a staggered manner is used to create mixing. The interactive vortices, i.e., the constructive interference between two vortices, were quite significant in enhancing mixing efficiency.

**Figure 8:**
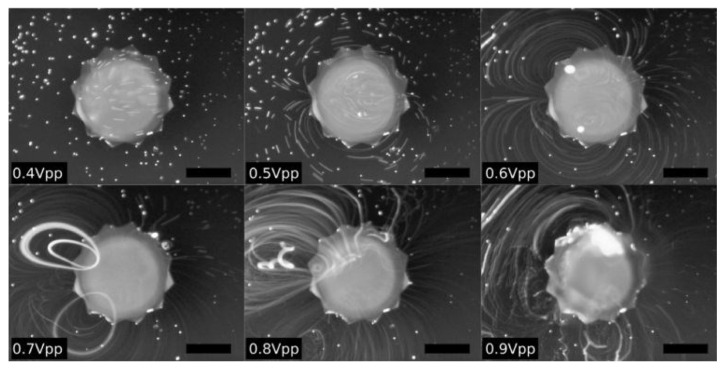
Visualisation of the streaming field induced by a variable actuation voltage amplitude swept from 0.4 to 0.9 $V_{p-p}$. Reproduced with permission from [93].

Flower-like sharp-edged structures have been investigated, with a study of the effects of spreading angle and tip angle [92]. Le et al. developed an ultrafast star-shaped micromixer using a silicon-based micromechanical oscillator to guide fluid in and out of the system, with mixing efficiency of 91% and low mixing time, as shown in Figure 8 [93].

Another interesting technique involves using ultrasound to induce mixing by applying it to bubbles formed in the bulk, known as microstreaming [94]. Microstreaming, specifically Rayleigh streaming, is caused by shear viscosity in the thin Stokes boundary layer near a solid boundary. The bubble is found to provide high efficiency as an element that propels the fluid element with characteristic velocity proportional to the square of the amplitude of the vibration. For separate fluids, streaming is not yet active in the bulk, but as diffusion occurs leading to motion smearing non-homogeneity, the streaming forces expand into the bulk, leading to more effective mixing. A large number of studies are performed on quasi-2D plane cavitation microstreaming, although micro-particle trajectories create a very complex behavior, especially a strong deviation from plane dynamics in regions close to the microbubble surface. This has important implications in usage of acoustic microstreaming for mixing. The aforementioned observation has been studied by Marin et al. [95], which revealed that the planar streamlines are actually projections of the pseudo toroidal shaped stream surface. This illustrates the complexity of acoustic streaming and its enhanced efficiency in mixing. The influence of acoustic microstreaming on fluid flow and, thus, its micromixing action (through the formation of strong vortices), is governed by

(18) $$u_{s}\cdot \left(\nabla u_{s}\right)=-\frac{1}{\rho }\nabla p+\nu \left(\nabla^{2}u_{s}\right)-F_{s}$$

(19) $$F_{s}=\frac{1}{2}Re\left[\langle \left(u_{a}\cdot \nabla \right)u_{a}\rangle \right]$$

where **u_s_** is the streaming velocity, **u_a_** is the complex amplitude of fluid vibration induced by the piezoelectric transducer, *p* is the pressure, ρ is the fluid density, ν is the kinematic viscosity, and **F_s_** is the force generated by the streaming flow induced from the first order oscillatory field. The characteristics and physical models of regime-wise vortices are discussed in depth in [96,97].

Conde et al. propose a hybrid micromixer that combines a soft slab with small packets over a hard substrate [40]. Ultrasound waves generate oscillations on trapped bubbles in the grooves, producing a strong microstreaming effect in the mixing chamber. Geng et al. present a numerical study on microstreaming, investigating different vortex patterns formed for varying channel aspect ratios and the impact of inlet velocity on mixing efficiency using Reynolds stress and low velocity methods [98].

Microstreaming has also been utilized to enhance droplet mixing in electrowetting microfluidic platforms. Won et al. inject bubbles into merged droplets and acoustically excite them to generate microstreaming patterns, significantly reducing the time required for droplet mixing [99]. The bubbles are later eliminated by activating a nearside electrode. It is important to note that microstreaming has also been demonstrated at higher frequencies where acoustic wavelength approaches the dimensions of the channel [97,100,101].

**Figure 9:**
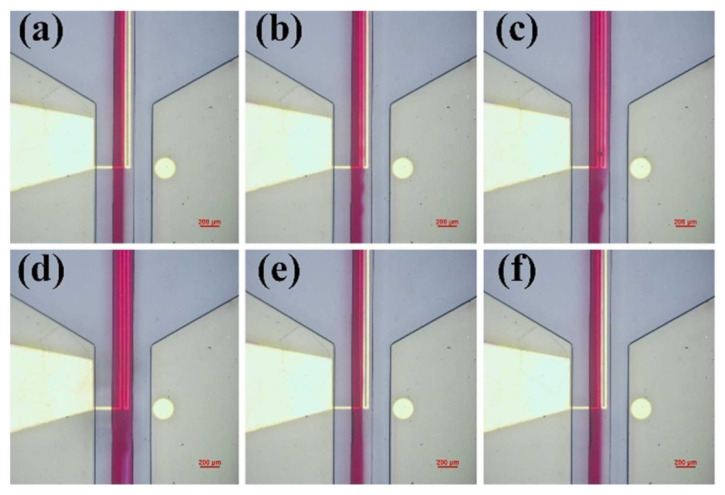
Surface acoustic wave (SAW) micromixing performance in a channel for an excitation frequency of (**a**) 17.1 MHz; (**b**) 17.4 MHz; (**c**) 17.8 MHz; (**d**) 18.2 MHz; (**e**) 18.6 MHz; (**f**) 19 MHz. The focused interdigital transducer (FIDT) has a resonance frequency of 18.2 MHz. Reproduced with permission from [102].

In addition to microstreaming, bulk acoustic waves (BAW) and surface acoustic waves (SAW), as presented in Figure 9, have been used to create perturbations in the fluid medium for enhanced mixing [102,103]. Mei et al. review the handling of SAW in acoustofluidics [104]. Overall, acoustic-driven micromixing offers diverse approaches and has shown promise in achieving efficient mixing in microfluidic systems.

### 3.4. Other Techniques

Droplet mixing is a unique technique that has been studied for efficient mixing in micromixers. Sakurai et al. present a novel micromixer where immiscible droplets are inserted into the mixing channel [105]. The geometry of the channel and the injection frequency of droplets play a role in the mixing performance. The droplets perturb the sample-buffer interface and enhance the mixing efficiency as they flow through the mixing channel.

In addition to electric potential and acoustics, other excitation sources can be utilized to induce disturbances in the fluid and achieve mixing. One such source is the magnetic force. Magnetohydrodynamics (MHD) is an interesting field to explore in this context. Bahrami et al. study the effect of a non-uniform magnetic field on a sinusoidal micromixer, demonstrating improved mixing quality with the use of a ferrofluid [38]. The distance between the magnet and the channel affects mixing efficiency. However, it should be noted that increasing magnetic field does not always enhance mixing and can have negative effects in certain cases. The disposition of magnets in the micromixer has also been studied to assess its effect on mixing efficiency [106].

There have been successful studies combining magnetic and droplet-based techniques to perform mixing in microchannels [107,108]. These mixers benefit from both the secondary bulk flow induced by the external magnetic field on a ferrofluid (magneto-convection) and the shear-driven circulating flow of the droplets. Optimizing the proportion of magnetic and shear forces is crucial for achieving optimal mixing, as dominance of either force can adversely affect efficiency.

Direct thermal energy can also be utilized in micromixing [109,110,111]. Increasing the temperature of the fluid bulk enhances diffusivity, and microheaters can be embedded in a channel to introduce thermal energy. Thermal bubbles are known to increase mixing efficiency. The temperature gradient formed in the channel generates convective secondary flow, creating an environment conducive to mixing. Recent studies have also focused on thermal mixing and the thermal behavior of non-Newtonian fluids [112,113].

Overall, these techniques highlight the diverse range of methods that can be employed for effective micromixing, utilizing droplets, magnetic forces, and thermal energy as additional driving forces to induce mixing in microchannels.

Table 2 summarizes the entire set of microfluidic mixers studied in this article.

## 4. Critical Assessment of Micromixing Techniques

Micromixers play a pivotal role in microfluidic systems, enabling efficient blending of fluids on a minute scale. As has been described in the prior section, various techniques have been developed to enhance micromixing, each with its unique advantages and complexities.

Geometrical micromixers capitalize on intricate channel designs to induce specific perturbations and fluid–fluid interface disruptions, either locally or globally. Rooted in fluid mechanics principles, these mixers deliver exceptional efficiency while maintaining a simplified manufacturing process. However, they usually necessitate external pumps for fluid propulsion. The combination of geometrical micromixers with other techniques holds promise for outstanding performance. Yet, integrating these methods can be challenging due to the non-linearity and non-planarity of designs, which may hinder the application of electric fields, ultrasonic waves, and other driving forces. Researchers are continuously innovating to strike a balance between complexity and feasibility, creating adaptable devices for lab-on-a-chip (LoC) applications.

Electrohydrodynamics stands out as a dominant force in the microfluidics realm, dictating mixing and instabilities. By manipulating parameters like voltage, phase difference, frequency, and electrode positioning, it is possible to exert impressive control over microchannel flows. The gamut of electrokinetic phenomena—electroosmosis, electrothermal effects, and electrophoresis—can be harnessed to modulate fluid behavior, contingent on factors such as fluid conductivity and thermochemical properties. While offering streamlined design possibilities compared to passive micromixers, this technique comes at the cost of elevated power consumption and possible elevated temperatures within the microchannel. Notably, electrohydrodynamic methods can be extended to poorly conductive substances by introducing inert metallic micro-particles, rendering them responsive to electric fields.

As for acoustofluidics, it provides an elegant means of achieving mixing without provoking electrochemical reactions. This technique proves invaluable when a localized actuator is needed to generate widespread effects. Nonetheless, it often necessitates auxiliary elements to amplify or propagate the induced effect throughout the microchannel. Integrating these elements to create microbubbles for microstreaming or protrusions for vibration amplification, can be intricate. However, unlike electroosmosis where the ionic concentration significantly influences the outcome, acoustofluidics remains indifferent to the fluid’s chemistry. Other techniques like magnetohydrodynamics are suitable for specific fluid types. Conductive fluids exposed to electric and magnetic fields can develop potential differences due to electromotive forces. A plethora of phenomena awaits exploration within microchannels due to their flexibility, robustness, and high surface-to-volume ratio. Precise understanding of fluid properties, chemical characteristics, and a strategic trade-off between complexity and efficiency become pivotal for successful application.

Choosing the most suitable micromixing approach depends on a multitude of factors that span from technical considerations to application-specific requirements. Here, we delve into the pivotal factors that guide the selection process when opting for a particular micromixing technique.

### 4.1. Fluid Characteristics and Chemistry

The inherent properties of the fluids being mixed significantly influence the choice of micromixing technique. Electro-osmotic methods, for instance, are highly sensitive to fluid conductivity and ionic composition. If the fluid is non-conductive or poorly conductive, employing electrodes loaded with inert micro-particles can extend the applicability of electrohydrodynamics. Acoustofluidic approaches, on the other hand, are relatively agnostic to fluid chemistry, making them suitable for a broader range of substances.

### 4.2. Required Mixing Efficiency

Different applications demand varying levels of mixing efficiency. Geometrical micromixers, despite their simplicity, might fall short in scenarios requiring rapid and precise mixing. In such cases, electrohydrodynamics and acoustofluidics exhibit higher potential due to their ability to generate intricate fluid dynamics that accelerate the mixing process. Bayareh et. al list micromixers of various types along with their mixing efficiencies, which highlights that it is possible in some cases even for geometric micromixers to reach efficiency in excess of 90% [114]. Nonetheless, the trade-off between efficiency, mixing length, and Re regime must always be considered in order to enact an optimal selection.

### 4.3. Energy Consumption and Heating

Energy consumption and the potential for heating within the microchannel are critical factors. Geometrical micromixers generally rely on passive mechanisms, resulting in lower energy requirements. Electrohydrodynamics, while offering fine control over mixing, can lead to elevated power consumption and a rise of temperature within the channel. Acoustofluidics, with its focus on non-contact phenomena, usually have a lower impact on energy consumption than electrohydrodynamics. Analyzing and optimizing power consumption becomes an important element when researchers design active micromixers, which acts as a significant factor in the overall figure of merit [115]. Depending on the criteria of interest, it may be necessary to take into account the energy consumed to actuate the fluid pumping action if applicable. Indeed, passive micromixing generally relies on a fluid flow, which may entail significant indirect power consumption, especially for complex geometrical shapes with high fluidic resistance.

### 4.4. External Element Integration

The feasibility of integrating external elements, such as electrodes, acoustic transducers, or magnets, plays a crucial role. Acoustofluidics might necessitate the incorporation of microbubbles or protrusions for efficient mixing, adding a layer of complexity to the design. Electrohydrodynamics, on the other hand, requires careful positioning of electrodes and consideration of electric field parameters. Geometrical approaches, obviously, are not affected by this concern.

### 4.5. System Complexity and Manufacturing

The complexity of the chosen approach and the ease of manufacturing are critical considerations. Geometrical micromixers, due to their simplified designs, generally offer straightforward manufacturing processes. Electrohydrodynamics and acoustofluidics often involve more intricate setups, potentially requiring advanced fabrication techniques or specialized components. Furthermore, one should not neglect the need for additional control electronics to drive any actuation element, and the resulting impacts on system integration. Nonetheless, passive micromixers, in order to provide efficiency comparable to active micromixers, are often formed of intricate geometries, so as to generate strong vortices and secondary flows as in spiral [19], serpentine [22] and related works on toroidal and helical channels [65]. These non-conventional channel shapes may entail non-standard lithographic and process steps, which also need to be assessed in terms of technological complexity.

### 4.6. Application-Specific Requirements

Applications often demand a specific level of control over spatial distribution and temporal evolution of mixing. Electrohydrodynamics excels in providing precise control over fluid movement through electrode manipulation. Acoustofluidics, meanwhile, generate spatial perturbations that propagate through the microchannel, inducing widespread mixing effects.

### 4.7. Spatial and Temporal Control

Ultimately, the choice of micromixing technique is driven by the specific requirements of the application at hand. For instance, if localized mixing within microdroplets is needed, acoustofluidics might be more suitable. On the other hand, if mixing biofluids is the goal, careful consideration of potential heating effects in electrohydrodynamics becomes paramount.

In short, the selection of a micromixing approach is a multifaceted decision, shaped by factors ranging from the inherent properties of the fluids to the intricacies of the application. Each technique offers a unique blend of advantages and constraints, demanding a comprehensive evaluation of these factors to determine the most fitting approach for achieving efficient and effective micromixing.

## 5. Conclusions

The pursuit of efficient micromixing in the realm of microfluidics has led to a diverse array of techniques and approaches. This review paper has explored the intricacies of several prominent methods, each with its own set of advantages, intricacies, and limitations.

The choice of the most suitable micromixing approach hinges on multifaceted considerations. Fluid characteristics, mixing efficiency requirements, energy consumption, external element integration, and application-specific needs all play a vital role in determining the most fitting technique. A delicate balance between complexity and efficiency must be struck, guided by a thorough understanding of the physical and chemical properties of the fluids involved and the application targeted.

### 5.1. Summary of Micromixing Techniques

Geometrical micromixers, with their elegant designs and simplicity, leverage fluid mechanics to create perturbations that trigger mixing. While cost-effective and easy to manufacture, these mixers excel when combined with other techniques to amplify their performance. Electrohydrodynamics stands out as a versatile powerhouse in micromixing. With its intricate interplay of electro-osmotic, electrophoretic, and electrothermal phenomena, it offers precise control over fluid movement, albeit at the cost of energy consumption and potential heating. The selection of electrodes, frequency, and positioning demands careful consideration to harness the full potential of this approach. Acoustofluidics, free from inducing chemical reactions and responsive to localized external sources, presents an appealing alternative. Leveraging acoustic waves to induce microstreaming, these methods enable efficient mixing without the need for direct physical contact. However, they often necessitate the incorporation of auxiliary elements, presenting design and integration challenges. Magnetohydrodynamics and thermal techniques have carved their own niche in the micromixing landscape. Magnetic fields play a pivotal role in mixing ferrofluids, while thermal gradients in channels bring about convection-driven mixing. These techniques often excel in specific scenarios, bringing their unique advantages to bear.

### 5.2. Future Developments

The field of micromixing continues to expand with the integration of various methods and technologies. Machine learning models are beginning to be utilized for optimizing channel dimensions and input parameters, thereby enhancing the efficiency of micromixers [116,117]. There are also possibilities for developing active micromixers with feedback control systems, potentially incorporating sensors (e.g., optical) for improved reliability and robustness [118].

In the wide application field of micromixing, each technique aims to provide particular advantages and specificities in the pursuit of efficient and effective fluid blending. As the field continues to advance, innovations in microfabrication, understanding of fluid dynamics, and integration of multi-technique approaches will undoubtedly further enrich this vibrant landscape.

## Figures and Tables

**Table 1:** Parameters governing the microscale flow. (ρ—fluid density; u—fluid velocity; L—characteristic length scale; D—fluid diffusivity ; μ—fluid viscosity; σ—surface tension; λ—mean free path; $\dot{\lambda }$—shear rate; τ—relaxation time; $\tau_{p}$—observation time).

Parameter	Expression
Reynolds (*Re*)	*ρ uL/μ*
Weissenberg (*Wi*)	$\tau \dot{\lambda }$
Elasticity (*El*)	*Wi/Re*
Deborah (*De*)	$\tau /\tau_{p}$
Péclet (*Pe*)	*uL/D*
Capillary (*Ca*)	*μu/σ*

**Table 2:** Summary of the micromixers reviewed in this article.

Micromixer Class	Type	Particularities	Reference
Geometry-assisted	Split and recombine	Fabricated using low cost X-Ray litho. MI = 96% at Re = 0.1.	[46]
	Split and recombine	Injection, recombination & zigzag combo.	[47]
	Split and recombine	Optimized using Taguchi/Grey analysis.	[48]
	Hydrodynamic focusing	Pulsed species mixing inducing Taylor–Aris dispersion.	[42]
	EC Cavity array	Surface ridges inducing a secondary flow.	[51]
	EC Cavity array	Staggered herringbone micromixer.	[52,53,54]
	EC Cavity array	Slanted grooves for spiral flow.	[55]
	Chaotic advection	Twisted channels fabricated using scaffold method.	[59]
	Serpentine	Elliptical curves to cause Dean vortices.	[22]
	Dean flow	Mixing action through Dean instability.	[62]
	Dean flow	Study of channel curve and spiraling impacts.	[63]
	Dean flow	Study of channel shape transition from toroidal to helical.	[65]
Electrokinetic	Electro-osmotic flow based	For non-Newtonian fluids.	[72,73]
	Electro-osmotic flow based	For viscoelastic fluids.	[74]
	ACEO Flow	Staggered electrodes causing transverse flows.	[45]
	ACEO Flow	Rubik’s cube-like module resembling within microchannel.	[75]
	ACEO Flow	Cantor Fractal microchannels under varying electric fields.	[76,77]
	ACET	Sequential mixing of three fluids by asymmetric vortices.	[81]
	ACET	AC Film heating. MI = 90%.	[83]
Acoustofluidic	Acoustic	Bubble-based.	[84,85,86]
	Acoustic	Solid-based.	[87,88]
	Microsolid	Sharp-edge structure generates centrifugal forces.	[90]
	Microsolid	Vortex constructive interference by staggered structures.	[91]
	Microsolid	Flower and star-shaped structures.	[92,93]
	Microstreaming	Acoustic waves activating trapped air bubbles.	[40]
	Microstreaming	Boundary-driven streaming flows.	[98]
	Microstreaming	Droplet-injected bubble.	[99]
	Microstreaming	Acoustic excitation for channel-sized wavelengths.	[97,100,101]
	Surface acoustic wave	Study of acoustothermal effects in response to applied signals.	[102]
	Surface acoustic wave	Single interdigital transducer. MI > 90% at flow of 200 μL/min.	[103]
Miscellaneous	Droplet injection	Study of diffusion coefficient and confluence angle effects.	[105]
	Magnetohydrodynamic	Magnets to enhance ferrofluid mixing in sinusoidal channels.	[38]
	Thermal	Temperature-enhanced diffusion.	[109,110,111]

## Data Availability

Not applicable.
